# Performance evaluation of the nano-biodegradable drilling fluid using the greenly synthesized zinc nanorods and *gundelia* seed waste

**DOI:** 10.1007/s11356-024-34561-7

**Published:** 2024-08-07

**Authors:** Jagar A. Ali, Rayan Gailani, Abdullah D. Abdullah, Pshtiwan T. Jaf, Sherwan Mohammed Simo, Mardin Abdalqadir, Vinos Mushir Faris

**Affiliations:** 1 Department of Petroleum Engineering, Faculty of Engineering, Soran University, Soran, Kurdistan Region Iraq; 2 Department of Geology, Palacký University, 17. Listopadu 12, Olomouc, 77146 Czech Republic; 3 Petroleum Pulsar, Erbil, Kurdistan Region Iraq; 4 Department of Petroleum Engineering, Al-Kitab University, Altun Kupri, Kirkuk, Iraq; 5 Department of Petroleum Engineering, Faculty of Engineering, Koya University, KOY45, Koya, Kurdistan Region Iraq; 6 Department of Petroleum Engineering, College of Engineering, University of Zakho, Zakho, Kurdistan Region Iraq; 7 Department of Engineering, School of Computing, Engineering and Digital Technologies, Teesside University, Middlesbrough, TS1 3BA UK; 8 Scientific Research Center, Soran University, Soran, Kurdistan-Region Iraq

**Keywords:** Nanorods, Biodegradable, Drilling fluid, Rheology, Sustainability, Fluid loss

## Abstract

Addressing the increasing demand for green additives in drilling fluids is essential for the sustainable development of the oil and gas industry. Fluid loss into porous and permeable formations during drilling presents significant challenges. This study introduced an innovative, environmentally sustainable drilling fluid known as nano-biodegradable drilling fluid (NBDF). The NBDF formulation incorporates greenly synthesized zinc nanorods (ZNRs) and *gundelia* seed shell powder, with ZNRs derived from *Cydonia oblonga* plant extracts using an eco-friendly method. The research developed multiple drilling fluid variants for experimentation: a reference drilling fluid (BM); biodegradable drilling fluid (BDF) with particle sizes of 75, 150, 300, and 600 µm at concentrations ranging from 0.5 to 1 wt% (GSMs); a drilling nanofluid (DNF) with ZNRs at a 0.1 wt% concentration (ZNR); and NBDF combining both nano and *gundelia* waste (GS-ZNR). Experimental tests were conducted under various temperature and pressure conditions, including low temperature and low pressure (LTLP) and high temperature and high pressure (HTHP). Rheological and filtration measurements were performed to assess the impact of the nano-biodegradable additives on flow behavior and fluid loss. Results indicated that incorporating 1 wt% of *gundelia* seed shell powder with a particle size of 75 µm led to a 19.61% reduction in fluid loss compared to BM at 75 °C and 200 psi. The performance of the same GSM improved by 31% under identical conditions when 1 wt% of zinc ZNRs was added. Notably, the GS-ZNR formulation demonstrated the most effective performance in reducing fluid loss into the formation, decreasing mud cake thickness, and enhancing the flow behavior of the non-Newtonian reference drilling fluid. This study highlights the relevance of particle size in the effectiveness of biodegradable additives and underscores the potential of NBDF to address environmental concerns in the oil and gas drilling industry.

## Introduction

**Fig. 1:**
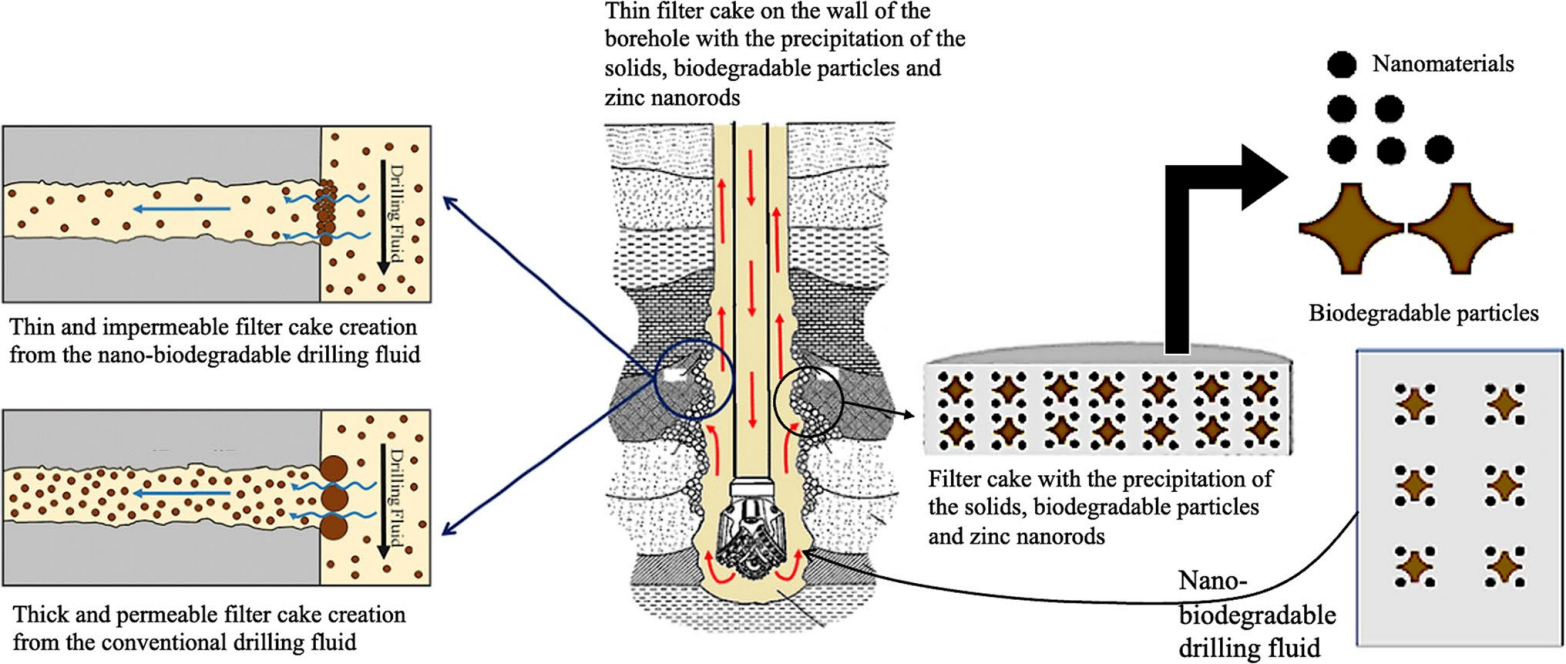
Schematic illustration of the formation of a filter cake on the borehole wall during the circulation of the nano-biodegradable drilling fluid (NBDF) (modified from Ali et al. 2022d, 2024a)

Issues within boreholes pose critical challenges in the gas and oil drilling process, with the potential loss of certain filtrates into subsurface formations during the circulation of drilling fluid within the borehole. This occurrence leads to escalated circulation rates and differential pressures between the wellbore and the fluid flow (Al-Yasir and Al-Sallami 2015; Tahr et al. 2023). Such fluid loss can cause severe complications, including pressure redistribution around the borehole, rock swelling, and alterations in rock strength (Davoodi et al. 2018; Li et al. 2023). Consequently, borehole stability is compromised, resulting in collapses, sloughing, and cavity formations (Iscan et al. 2007; Abed et al. 2022). These issues can be minimized by modifying the main properties of the drilling fluid, such as apparent viscosity, plastic viscosity, gel strength, yield point, filter cake, and fluid loss. As drilling fluid pass through the borehole and returns to the surface, it interacts with the formation rock (Ragab and Noah 2014; Al-Haj et al. 2022; Jameel and Ali 2023; Ali et al. 2024a). Biodegradable materials and nanomaterials within the fluid adhere to formation rock surfaces due to factors like electrostatic forces and Van Der Waals interactions (Ali et al. 2022a,b). Nanomaterials, outstanding to their substantial surface area and reactivity, create a stable coating on the borehole wall, enhancing its mechanical properties, reducing permeability, and mitigating wellbore instability issues (Fig. 1). Simultaneously, biodegradable compounds facilitate the breakdown of organic matter present in the drilling fluid, promoting environmentally conscious drilling practices (Shahbazi et al. 2020; Raza et al. 2023). Furthermore, the utilization of nano-biodegradable components reflects a promise to sustainable drilling methodologies and environmental preservation by leaving behind a favorable filter cake on the borehole wall which is impermeable and thin (see Fig. 1).

Biodegradable wastes and biopolymers were employed in the drilling process to improve the performance of water-based drilling fluids (WBDFs) in ecofriendly and sustainable way (Al-Hameedi et al. 2020; Sun et al. 2021; Ali et al. 2024a, b). The carboxymethyl chitosan biopolymer was added to the WBDFs, resulting in reduced yield point and filtrate volume entering the formation (Lei et al. 2020). Meanwhile, Ismail et al. (2020) employed *Rhizophora Mucronata Tannin* extract as a biopolymer additive, effectively diminishing gel strength and fluid loss volume to optimal levels using 8 gm of *Rhizophora Mucronata Tannin* extract. Al-Hameedi et al. (2019) incorporated grass powder as a biodegradable waste material to enhance drilling fluid properties. Their findings suggested a 42% reduce in fluid loss, accompanied by an improve in gel strength starting 22 to 26 Ib/100 ft2. In a separate study, Medved et al. (2022) observed significant reductions in filtration 61.54% in permeability plugging tester filtration and 42% in API filtration using an optimal concentration of 1.5 wt% mandarin peel. Recently, Awl et al. (2023) utilized the powder of broad bean peel, achieving a 35% drop in fluid loss by adding 3 wt% to the BM. Additionally, Raza et al. (2023) successfully enhanced drilling fluid rheological properties by using rice husk ash as sustainable additive. Nanotechnology has emerged as a tool to improve gas and oil drilling operations, particularly in addressing borehole issues by changing the fluid loss volume, thickness of the filter cake, plastic viscosity, yield point, and gel strength of circulating drilling mud (Vryzas et al. 2018; Ali et al. 2019; Li et al. 2019; Torres-Carrasco et al. 2019; Cheraghian 2021; Ibrahim et al. 2022; Bardhan et al. 2023a; Abdullah et al. 2024; Li et al. 2024a, b). Adding nanoparticles, typically silicon dioxide (NPs), enhances the transportation efficiency of drilling cuttings by adjusting the fluid’s rheological properties (Gbadamosi et al. 2019). Furthermore, Dejtaradon et al. (2019) highlighted significant improvements in rheological characteristics when using nano-formulated drilling fluids, with zinc nanoparticles exhibiting a more profound impact compared to copper nanoparticles. Zinc oxide (ZnO) nanostructures synthesized under microwave irradiation exhibit shapes like rods and flowers, significantly influencing the properties of WBDFs. The study found that ZnO nanoflowers enhance WBDF stability and filtration control, reducing HPHT filtrate volume by 56% and maintaining a consistent rheological profile, highlighting the impact of nanostructure morphology on drilling fluid performance (Prajapati et al. 2023). Industrially prepared silica nanoparticles coated with AEAPTS significantly enhance the rheology and filtration control of water-based drilling fluids at elevated temperatures. The addition of these coated nanoparticles reduced rheological degradation from 60 to 20% after thermal aging and improved viscosity and filtration properties, demonstrating their effectiveness as additives for high-temperature drilling applications (Bardhan et al. 2023b). Moreover, the introduction of bismuth ferrite nanoparticles increased the yield point and plastic viscosity values with the ratio of ¼ and reduced the fluid loss by 35% at ambient temperature (Perween et al. 2019).

This research aims to prepare sustainable modified drilling fluids with enhanced filtration characteristics using *gundelia* seed shell (GSM) and greenly synthesized zinc ZNRs. The zinc ZNRs, derived from *Cydonia oblonga* plant extract collected from the Kurdistan Region, were investigated for their effects on drilling fluid performance. Rheological and filtration measurements were conducted under varying pressure and temperature conditions to evaluate the impact of GSM and zinc ZNRs at different particle sizes and concentrations on drilling fluid performance.

## Material and methods

### Materials

Different additives were employed in this research. Bentonite with a purity 95% and a particle size between 0.8 µm and 2 µm, supplied by Carl Roth, was utilized to increase viscosity and weight. Soda ash was used to manage total hardness. A starch polymer with 95% purity (molecular weight MW of 343 g/mol), supplied by Carl Roth, was incorporated to improve filtration properties, and NaOH was introduced to raise the pH level. All these additives were sourced from Pulsar Petroleum Company. Zinc acetate, NaOH, and ethanol, each boasting a purity of 99%, were procured from Merck Company. These specific materials were integral in synthesizing zinc ZNRs, ranging in size from 1 to 100 nm. Additionally, *gundelia* seeds were gathered from a close field. After drying at ambient temperature, the *gundelia* seed shells underwent grinding into small particles using a grinder. Following this, the particles were sorted into different sizes 75, 150, 300, and 600 µm using sieves.

### Green synthesis of ZNRs

**Fig. 2:**
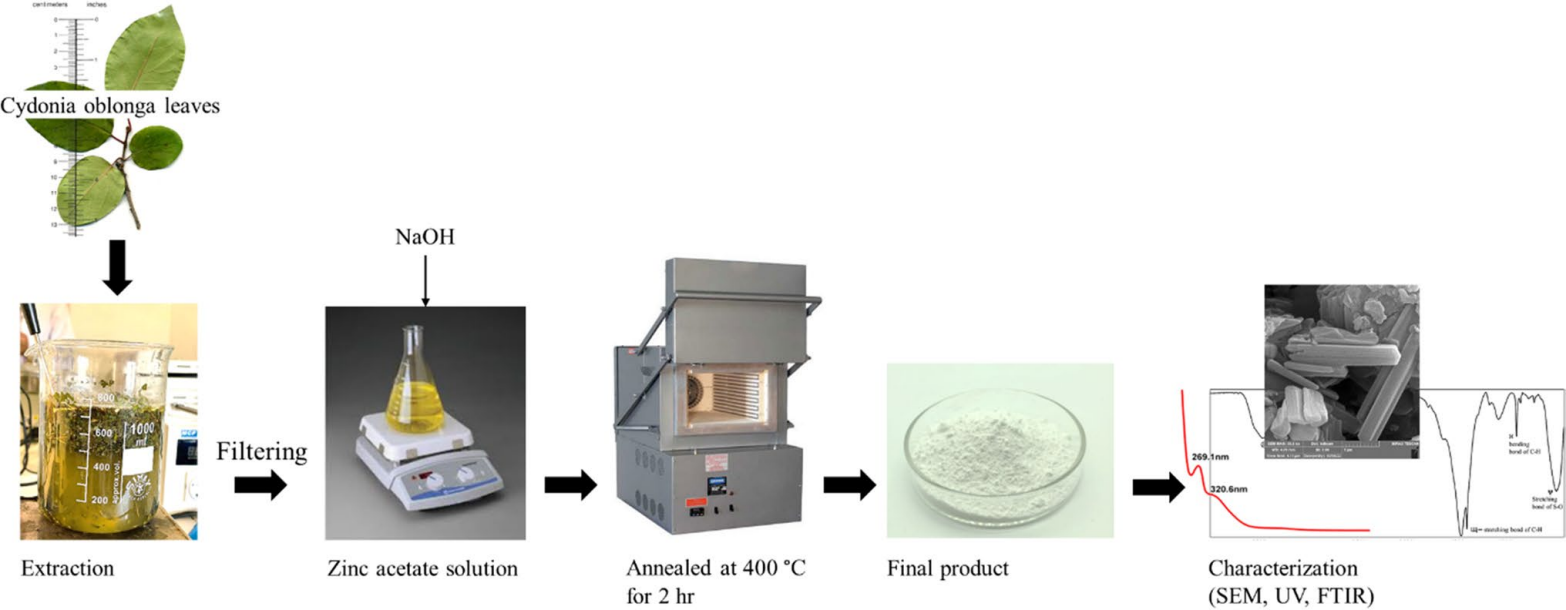
Schematic representation of ZNRs synthesis using the leaf extract of *Cydonia Oblonga*

The *Cydonia oblonga* plant leaves were cleaned using double-distilled water, dried, and cut into small pieces. Next, 10 g of the prepared leaf powder was placed into a flask with 100 mL of distilled water. Mixture was then heated for 40 min at 60 °C. Once cooling to room temperature, the extract underwent filtration using Whatman No. 1 filter paper to remove any unnecessary organic residues. Concurrently, 2 mg of zinc acetate dissolved in 50 mL of double-distilled water was stirred for 20 min at 80 °C. Then, 50 mL of the plant extract solution was gradually added to the dissolved zinc. This resulting mixture was placed on a hotplate, heated, and stirred at 70 °C for 45 min until it displayed a yellowish color, indicating the synthesis of ZNRs upon adding 50 mL of NaOH. The precipitate obtained was separated from the mixture through centrifugation at 7000 rpm for 20 min and subsequently subjected to heating at 400 °C for 2 h in an oven to eliminate impurities and organic materials surrounding the synthesized nanorods as shown in Fig. 2.

### Characterization of ZNRs

The UV–Visible spectra of ZNR_S_ were captured using a Super Aquarius Spectrophotometer-1000, covering a wavelength range of 200–700 nm with 1-nm resolution, with distilled water as the reference baseline, alongside comparative spectra for plant extract, sodium hydroxide, and zinc acetate dihydrate. Morphology analysis using an LVEM5 Benchtop Electron microscope was conducted through scanning electron microscopy (SEM), revealing essential details about the surface structures and size distributions of the ZNR_S_. Fourier transform infrared (FTIR) spectra obtained from a PerkinElmer Spectrum 100 FTIR, spanning 400 to 4000 cm^−1^ with a resolution of 1 cm^−1^, identified the chemical bonds and molecular structures, indicating possible organic coatings or functional groups. Dynamic light scattering (DLS) was used to analyze the particle size distribution, providing vital data on size uniformity and polydispersity, critical for various applications where consistent particle size is crucial.

### Preparation of the reference drilling fluid

BM primarily comprised water as the base, along with bentonite, and additional components for instance soda ash (Na_2_CO_3_), caustic soda (NaOH), and polymer as outlined in Table 1. The drilling fluid samples were prepared in accordance with the standard American Petroleum Institute (API SPEC 13A, 1993), following the procedure described by Ali et al. (2022c).

**Table 1:** BM composition

Materials	NaOH (g)	Water (mL)	Polymer (g)	Soda ash (g)	Bentonite (g)
Concentration	0.1	350	0.50	0.50	20

### Development of the biodegradable, nano and nano-biodegradable drilling fluids

Besides the BM, ten drilling fluid samples were prepared by incorporating various additives. These additives included ZNRs and a biodegradable powder derived from *gundelia* seed shells, integrated into the BM at various concentrations as specified in Table 2. Initially, the *gundelia* seed shell powder, functioning as the biodegradable additive, was employed to formulate biodegradable drilling fluids (BDFs) (GSMs) at concentrations of 0.05 and 0.10 wt% for four distinct particle sizes (75, 150, 300, and 600 µm). Following this, ZNR nanofluids were created by adding synthesized ZNRs at 0.1 wt% to the BM. Initially mixed with water for 2 h using an ultrasonic homogenizer, these ZNRs were later blended with bentonite and other fluid components for 1 h using a Hamilton Beach Commercial mixer. Furthermore, 0.1 wt% of zinc ZNRs were incorporated into BDFs to generate GS-ZNR NBDFs.

**Table 2:** Composition of biodegradables (GSM), nano (ZNR), nano-biodegradables (GS-ZNR) drilling fluids

Drilling fluid component	Drilling fluid	Biodegradable drilling fluid								Nano-drilling fluid	
		75 µ		150 µ		300 µ		600 µ		Zinc ZNRs	
		1	2	1	2	1	2	1	2	1	2
Bentonite, g	20	20	20	20	20	20	20	20	20	20	20
Water, mL	350	350	350	350	350	350	350	350	350	350	350
Soda ash, g	0.50	0.50	0.50	0.50	0.50	0.50	0.50	0.50	0.50	0.50	0.50
Polymer, g	0.50	0.50	0.50	0.50	0.50	0.50	0.50	0.50	0.50	0.50	0.50
NaOH, g	0.10	0.10	0.10	0.10	0.10	0.10	0.10	0.10	0.10	0.10	0.10
*Gunelia seed* peel, wt%	–	0.50	1.0	0.50	1.0	0.50	1.0	0.50	1.0	–	1.0
Zinc ZNRs, wt%	–	–	–	–	0.1	–	–	–	–	0.1	0.1

### Drilling fluid testing

**Fig. 3:**
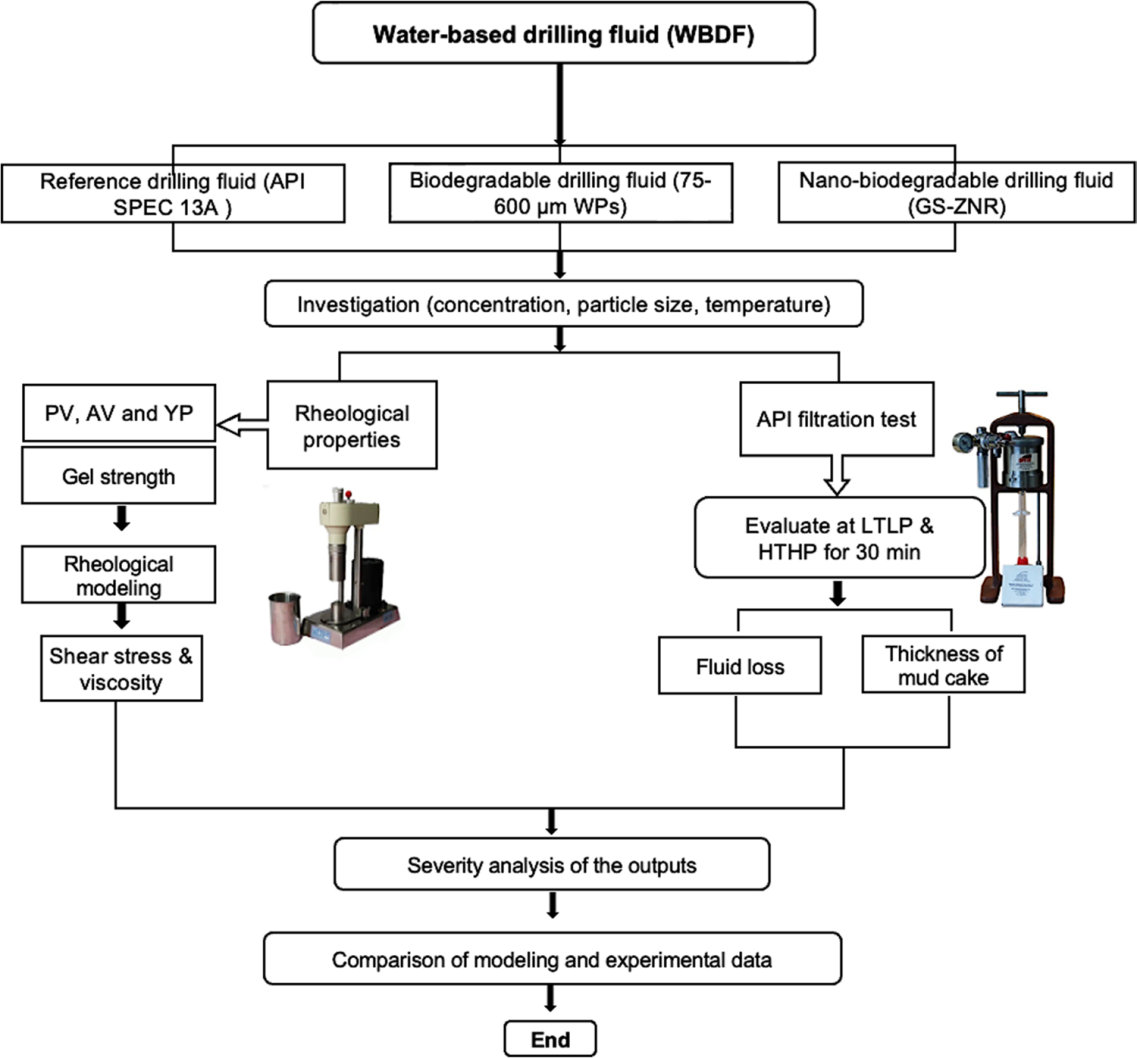
Diagram illustrating the sequence of procedural steps

Figure 3 illustrates a flowchart depicting the experimental steps conducted in this study. The study commenced with the collection and preparation of materials and additives, encompassing conventional additives, gundelia seed shell, and ZNRs (synthesis and characterization). These materials were subsequently employed in formulating the BM, drilling nanofluid (DNF), BDF, and NBDF. Following this, drilling fluid testing, involving rheological and filtration measurements, was conducted under LTLP and HTHP conditions. The collected experimental data were analyzed using the rheological model.

#### Rheological properties

The apparent viscosity, yield point, plastic viscosity, and gel strength (initial at 10 s and final at 10 min) as the main effective rheological characteristics of all formulated samples were assessed utilizing the FANN 35 viscometer at 50 °C aligns with the standard temperature as per API Specification 13A (1993). Configuration of the viscometer cup was done with the BM, followed by adjusting the equipment settings accordingly. Activation of the gear switch initiated varying rotational speeds of the rotor. The maximum deflection of the dial before gel breakdown was measured at 10 s and 10 min to estimate the initial and final gel strengths of the fluids, respectively. Meanwhile, the necessary measurements of the viscometer response for the computing the viscosity and yield point were taken at different speeds of the rotation (low speed 3 and 6 RPM, moderate speed 100 and 200 RPM, and high speed 600 and 300 RPM) using Eqs. 1–3 (Madu et al. 2024).

1 $$Apparentviscosity\mathit,\mathit\;\mu a\mathit\;\left(cP\right)\mathit\;\mathit=\mathit\;\mathit{600}\mathit\;rpm\mathit\;reading\mathit/\mathit2$$

2 $$Plasticviscosity\mathit,\mathit\;\mu p\mathit\;\left(cP\right)\mathit\;\mathit=\mathit\;\mathit{600}\mathit\;rpm\mathit\;reading\mathit-\mathit{300}\mathit\;rpm\mathit\;reading$$

3 $$Yieldpoint\mathit,\mathit\;\tau y\mathit\;\left(\frac{Ib}{\mathit{100}}ft^{\mathit2}\right)\mathit\;\mathit=\mathit\;\mathit{300}\mathit\;rpm\mathit\;reading\mathit-\mu p$$

In addition, the relationship between the shear rate and shear strain is also estimated using different rheological models such as Power law, Weibull, Herschel-Bulkley, and Vipulananda (Tahr et al. 2022a, b). In accordance with API recommendations (Bigham model), the Fann viscometer and computation of shear stress are determined by the formula (Ali et al. 2024a, b):

4 $$\text{Shear stress}={\text{K}}_{1}{\text{K}}_{2}\uptheta$$

where ϴ is viscometer reading, *K*_1_ is torsion constant of 386 dyne-cm/degree deflection, and K_2_ is shear stress constant equal to 0.01323 cm^−2^. Then the shear rate can be calculated as follow (Ali et al. 2024a).

5 $$\text{Shear rate }={\text{K}}_{3}\text{N}$$

where *N* is the viscometer speed in rpm and *K*_3_ is the shear rate constant of1.7023 S^−1^.

6 $$\text{Apparent viscosity}=\frac{\text{shear stress}}{\text{shear rate}}\times 100=\frac{\uptau }{\upgamma }\times 100$$

#### Filtration properties

The filtration properties were studied for all the prepared drilling fluid samples. This investigation was conducted under two distinct conditions: LTLP at room temperature and 100 psi, and HTHP at 75 °C and 200 psi as given by API Specification 13A (1993). The assessment duration for each measurement was set at 30 min. Initiating the Series 300 LTLP filter press setup involved placing a filter paper in the cell and introducing drilling fluid. The filtrate tube was then positioned over a graduated cylinder, and the filter press was configured before commencing the testing process with the initiation of the timer. At various intervals during the 30-min timeframe, the volume of filtrate in the graduated cylinder was noted along with the thickness of the filter cake (Table 3). For testing fluid loss and mud cake formation under extreme conditions, the HTHP API filter press, modified by the OFITE Company in Houston, USA, using model 170–01-1, was utilized. This specialized equipment was designed to assess the filtering capabilities of drilling fluid samples under reservoir-like conditions of 200-psi pressure and 75 °C temperature.

**Table 3:** Recorded measurements of rheological properties for NBDF, DNF and BDF and BM

Drilling fluid	Sample	Size (µm)	Conce (wt%)	YP (Ib/100ft^2^)	µ_p_ (cP)	µ_a_ (cP)	Gel strength (lb/100 ft^2^)	
							Gel_inital_	Gel_final_
Reference drilling fluid	BM			6.5	7	13	3	6
Biodegradable drilling fluid	GSM	75	0.5	3.5	4	7.5	2	5
	GSM		1.0	4	4	8	3	6
	GSM	150	0.5	4.25	4.5	8.75	2	6
	GSM		1.0	5.75	6	11.7	4	9.5
	GSM	300	0.5	9.5	7	16.5	8	16
	GSM		1.0	6.5	5.5	12	6	11
	GSM	600	0.5	14.5	12	26.5	30	46
	GSM		1.0	16.5	12	28.5	32	48
Drilling nanofluid	ZNR		0.1	7	13	3	13	17
Nano-biodegradable drilling fluid	GS-ZNR	75	0.1	9	7	11.5	5.5	10

## Results and discussion

### Characterization of the synthesized ZNRs

**Fig. 4:**
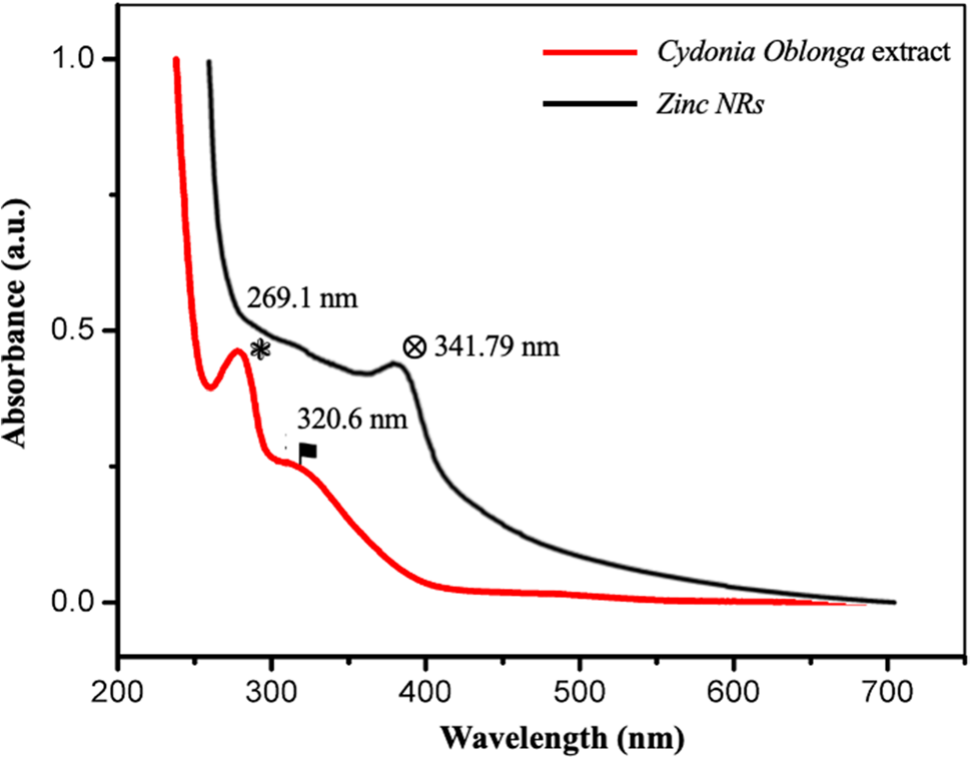
UV–vis spectra of the *Cydonia Oblonga* leaf extract and synthesized ZNRs

The eco-friendly synthesis of ZNRs led to subsequent characterization using SEM, UV–Visible spectroscopy, and DLS to ascertain their size and structure. The washed and dried nanorods were reconstituted in sterile deionized water to form a diluted suspension. UV–Visible spectra were captured using a UV–Vis double-beam spectrophotometer (Super Aquarius Spectrophotometer-1000) across the wavelength range of 200–700 nm with a 1-nm resolution. Before conducting spectral measurements, distilled water was utilized to set the reference baseline. Spectra for plant extract, sodium hydroxide, and zinc acetate dihydrate were also recorded for comparative analysis. Figure 4 displays the UV–Vis spectrum of the *Cydonia Oblonga* leaf extract juxtaposed with the nano-solution of the synthesized ZNRs. The peaks observed at 269.1 nm and 320.6 nm are likely associated with the phenolic components, specifically flavonoids and polyphenols, present in the *Cydonia oblonga* leaf extract. This extract predominantly contains flavonoids, polyphenols, and other phenolic compounds such as quercetin, kaempferol, and rutin, which are crucial in reducing zinc ions and stabilizing them at a nanoscale level. These phytochemicals wield significant influence over both the shape and size of the resulting nanorods. Additionally, the UV–Vis absorption spectrum confirms the presence of ZNRs at an ultraviolet wavelength of 341.79 nm.

**Fig. 5:**
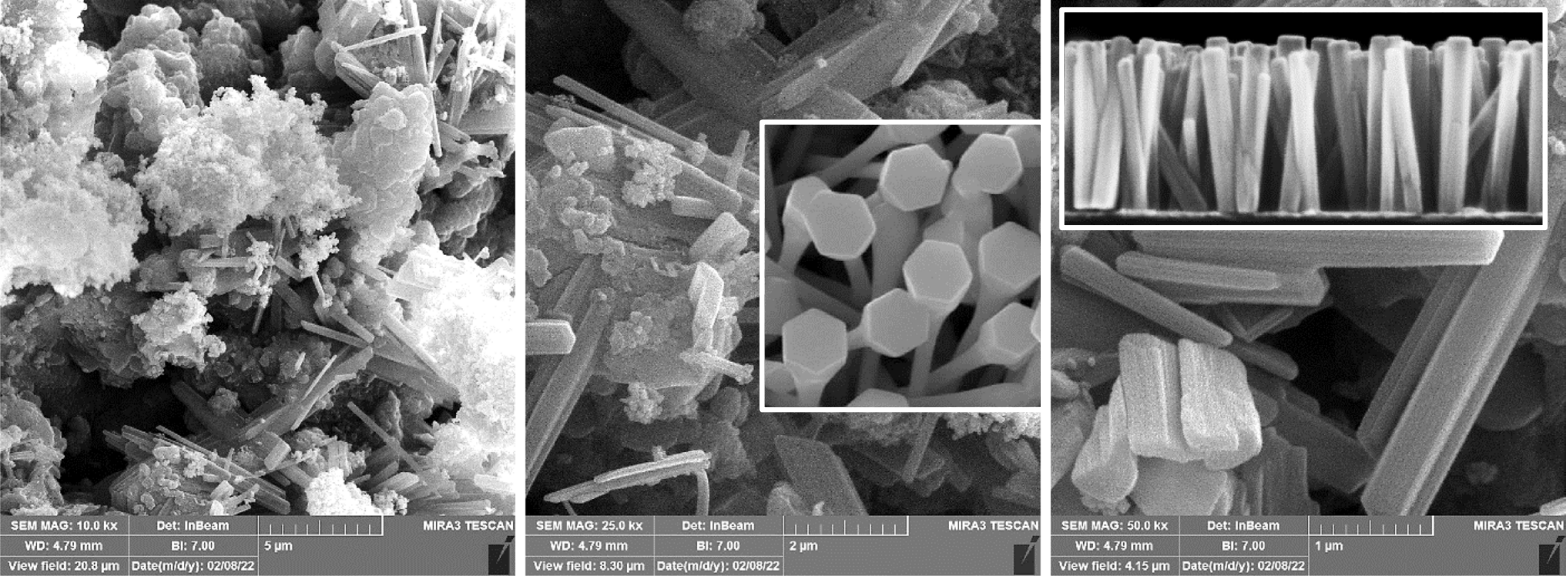
SEM of the synthesized zinc ZNRs at different scales from 1 to 5 µm

**Fig. 6:**
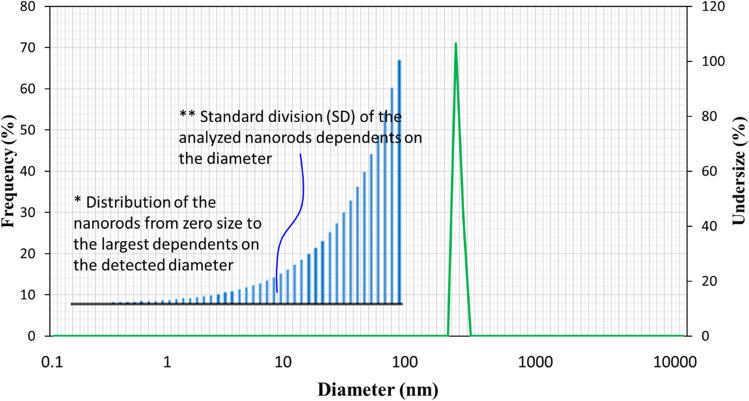
DLS analysis of ZNRs synthesized from *Cydonia Oblonga* extract

SEM was employed to observe the morphologies of the yellow layer. Figure 5 displays the SEM micrograph of the synthesized zinc ZNRs at various magnifications. The nanorods’ fabrication is evident from their cylindrical and hexagonal shape, each dimension measuring less than 50 nm. Meanwhile, Fig. 6 demonstrates the application of DLS, a usually used technique for determining the hydrodynamic diameter of nanoparticles by analyzing their Brownian motion within a suspension. Discrepancies in nanoparticle size may relate to the polydispersity index (PDI), reflecting the presence of nanoparticles in aggregates or agglomerates. The synthesized ZNRs exhibit an average size of approximately 31 nm. The variation in diameter measurements obtained via DLS and SEM stems from the different processes involved in sample preparation.

**Fig. 7:**
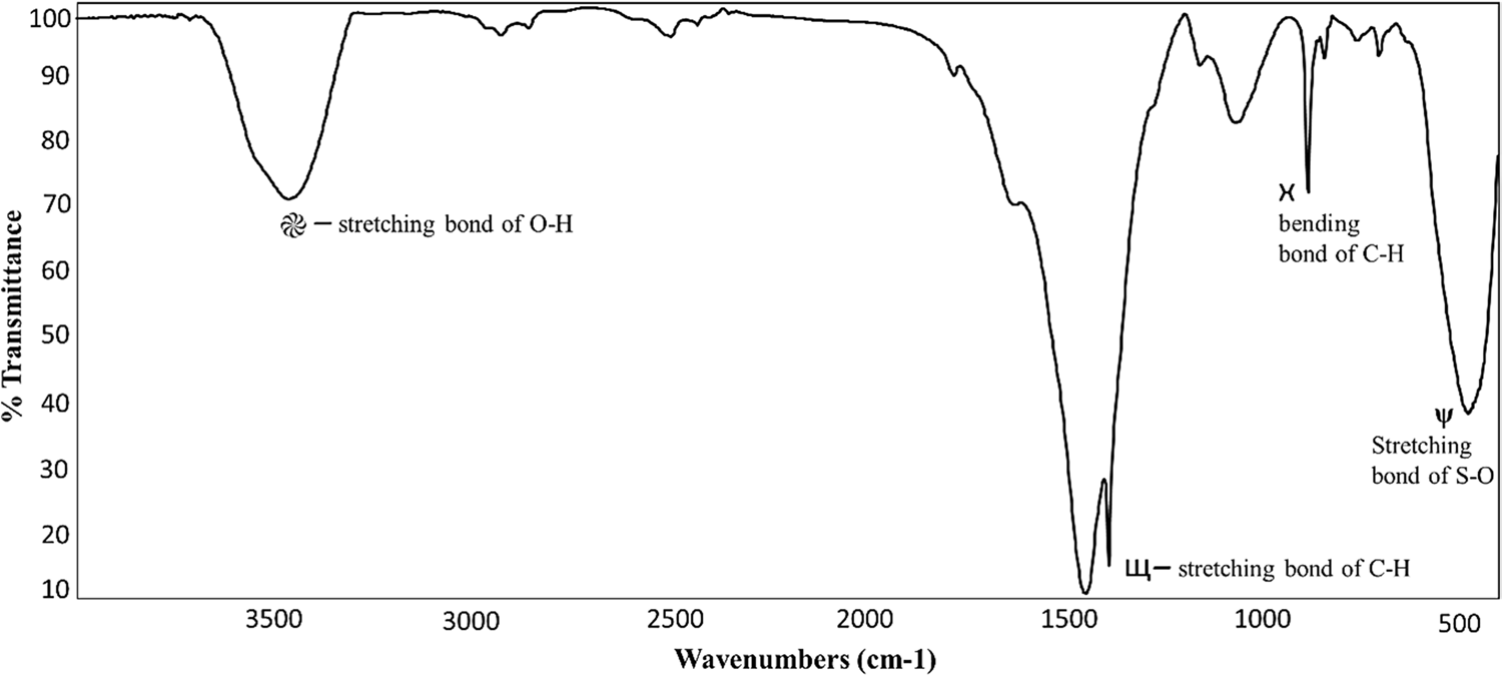
FTIR spectrum of the synthesized zinc ZNRs

The FTIR spectrum presented in Fig. 7 displays absorption peaks spanning from around 4000 to 400 cm^−1^, showcasing the biosynthesized ZNRs’ characteristics. Several absorption bands are visible, notably at 1075, 1250, 1382, 1595, 2922, and 3440 cm^−1^. These bands signify the presence of biomolecules crucial for the stabilization, capping, and reduction of ZnO nanorods. Specifically, the bands observed at 1075 cm^−1^ and 1250 cm^−1^ characterize C–C and C–N stretching, indicating the presence of protein amines. Meanwhile, the band at 1595 cm^−1^ is likely connected to C–N/C–C stretching vibrations of amines or alkene, and the 1382 cm^−1^ band signifies the O = N symmetry stretching characteristic of nitro compounds. At 2922 cm^−1^, the band is associated with C–H stretching vibrations of amines. Additionally, the 3440 cm^−1^ band indicates O–H stretching vibrations, suggesting the presence of phenol and alcohol.

### Rheological properties

Table 3 displays the findings of rheological properties for the prepared samples. In the case of the BM, the apparent and plastic viscosities measured 13 and 7 cP, respectively. The yield point was recorded at 6.5 lb/100ft^2^, accompanied by gel strengths of 3 lb/100ft^2^ at 10 s and 6 lb/100ft^2^ at 10 min. Overall, the behavior of the BM was adversely altered, notably influenced by the waste powder and synthesized ZNRs. The *gundelia* seed shell powder, particularly at a size of 600 µm, exerted the most substantial effect on the BM, while the impact of zinc ZNRs remained relatively minimal. However, the NBDF moderately influenced the rheological properties, contributing to an improvement within the desirable range.

- Biodegradable drilling fluid

**Fig. 8:**
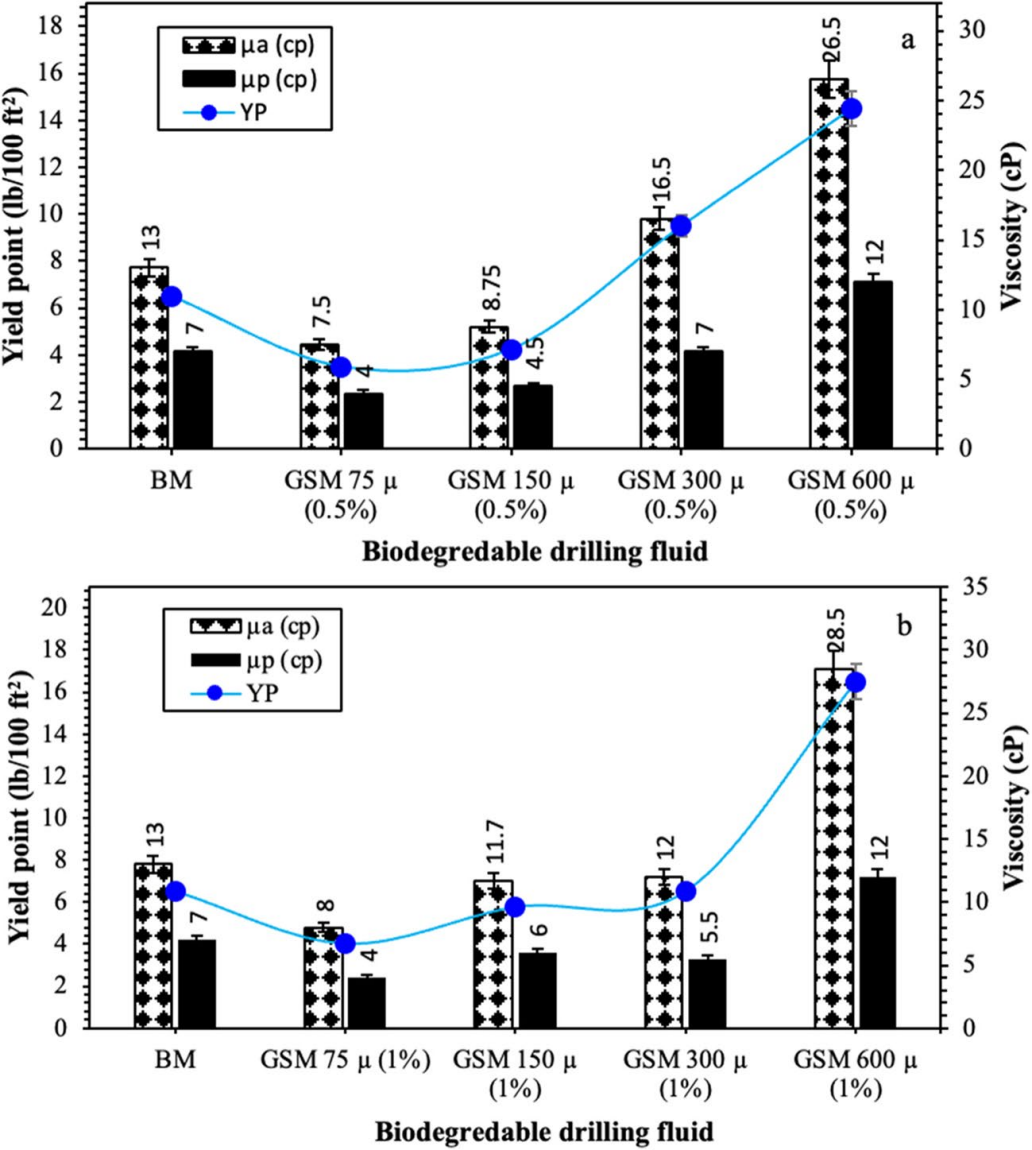
Plastic viscosity (µp), apparent viscosity (µa), and yield point of the BDFs prepared from mixing the *gundelia* seed shell at concentration of **a** 0.5 wt%, and **b** 1.0 wt%

**Fig. 9:**
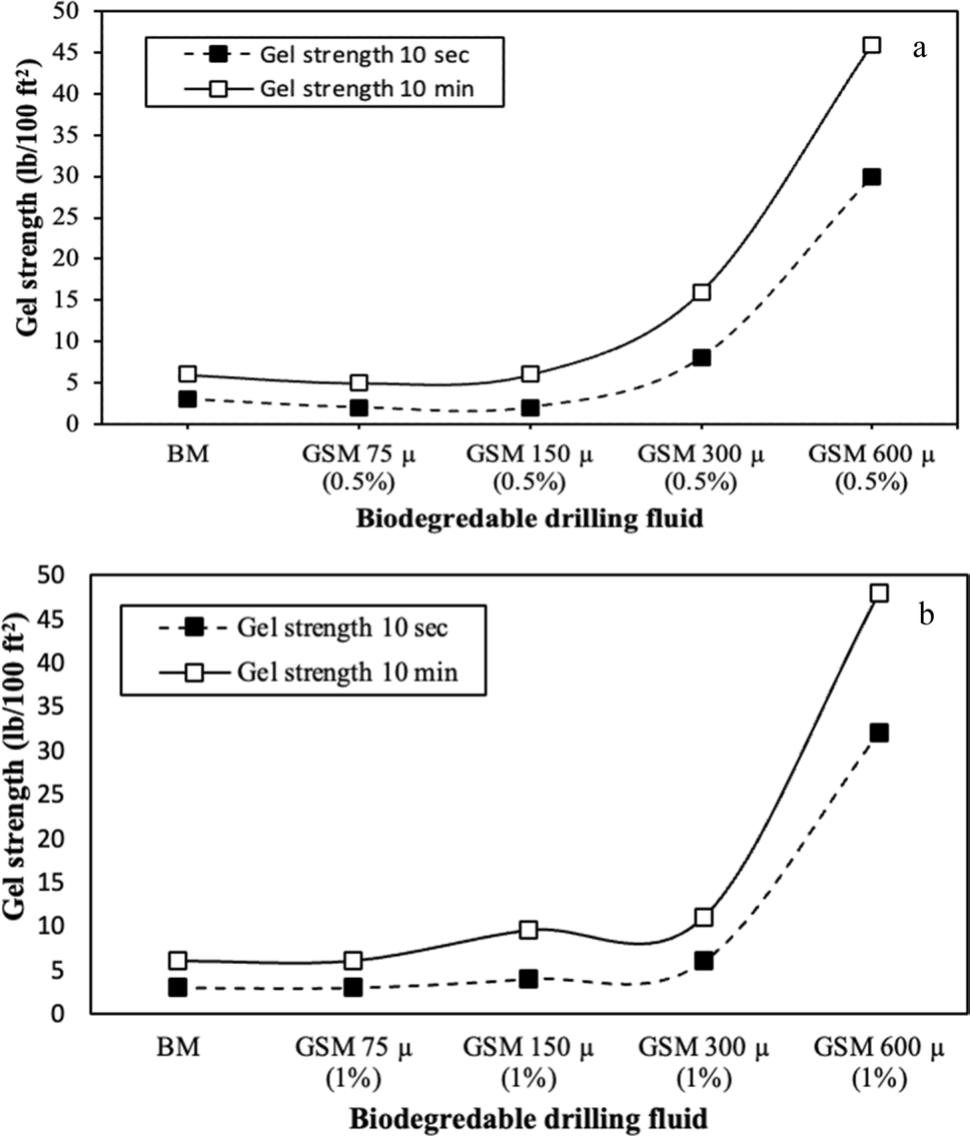
Gel strengths at (10 s and 10 min) of the BDFs prepared from mixing the *gundelia* seed shell at concentration of **a** 0.5 wt%, and **b** 1.0 wt%

The BDF incorporates environmentally friendly waste into the BM by introducing *gundelia* seed shell at various particle sizes. For the BDF formulated with a 0.5 wt% concentration of *gundelia* seed shell powder and a particle size of 75 µm, rheological properties were measured. These properties include a plastic viscosity of 4 cP, an apparent viscosity of 7.5 cP, and a yield point of 3.5 lb/100 ft^2^, as shown in Fig. 8a. Furthermore, the initial and final gel strength for the same drilling fluid were observed to be 2 and 5 lb/100 ft^2^, respectively (Fig. 9a). The reduction in rheological properties of *Gundelia* seed shell samples with smaller particle size can be primarily attributed to the interaction between the seed shell particles and the base mud components (Zhong et al. 2022). The fibrous and organic nature of *Gundelia* seed shells can interfere with the natural gel structure of the base mud, leading to a decrease in viscosity and gel strength. These natural fibers might absorb water from the drilling fluid, causing a reduction in the fluid’s overall viscosity (Ali et al. 2022b). Another contributing factor is the particle size and distribution of the *Gundelia* seed shell. The *gundelia* seed shell powder and a particle size of 75 µm can lead to changes in the fluid’s particle size distribution, which can disrupt the mud’s rheology (Nascimento et al. 2019). This disruption can decrease the yield point and plastic viscosity of the drilling fluid, as the particles may not interact as effectively as the finer clay particles in the base mud (Ghasemi et al. 2018). However, observations indicate that as the particle size increases, the rheological properties begin to escalate, reaching their peak values at a particle size of 600 µm. At this size, the yield point, apparent viscosity, and plastic viscosity attained their highest values of 14.5 lb/100 ft^2^, 26.5 cP, and 12 cP, respectively.

Furthermore, for the same GSM concentration and particle size, the initial and final gel strength measures 30 and 46 lb/100 ft^2^, correspondingly, as illustrated in Figs. 8a and 9a. Increasing the GSM concentration to 1 wt% results in a slight increase in the rheological properties. However, there is no change in plastic viscosity compared to the BDF containing 0.5 wt% GSM concentration. When 1 wt% GSM is added to drilling fluid with larger particle sizes, all rheological properties increase, reaching their peak values at the largest particle size of 600 µm (refer to Fig. 8b). Among the prepared BDFs, the highest apparent viscosity of 28.5 cP was achieved with GSM 600 µm (1%) formulated by mixing 1 wt% of 600-µm-sized *gundelia* seed shell powder with the BM. The plastic viscosity of the BDFs increases by 8 cP with the increase in particle size from 75 to 600 µm at both 0.5 and 1 wt%. Figure 9 demonstrates a significant influence of the *gundelia* seed shell powder’s particle size on the gel strength of the BDF. In Fig. 9a, both gel strength trends are showed for the BM and BDF at 0.5 wt% and particle sizes ranging from 75 to 600 µm. Notably, the initial gel strength values are lower compared to the final gel strength across all drilling fluid samples. There is a considerable overall increase of 1000% in gel strength values, elevating from 3–4.5 to 30–45 lb/100 ft^2^ when 0.5 wt% of *gundelia* seed shell powder with a particle size of 600 µm was added to BM. Figure 9b highlights a similar trend in gel strength observed in the BDFs formulated at a concentration of 1 wt%, under the influence of particle size. The behavior of gel strength remained consistent with the increase in particle size, mirroring the observed pattern in the 0.5 wt% concentration BDFs.

- Drilling nanofluid

**Fig. 10:**
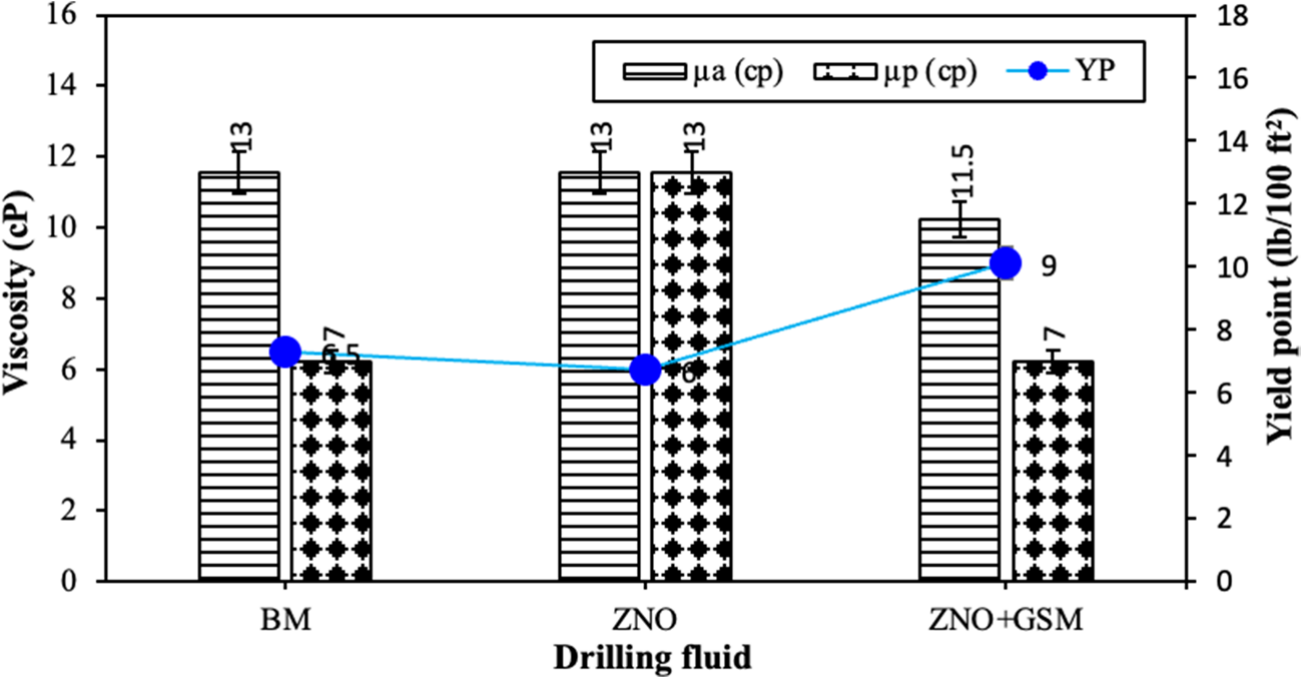
The findings of the BM, DNF, and NBDFs’ plastic viscosity (µp), apparent viscosity (µa) and yield point

**Fig. 11:**
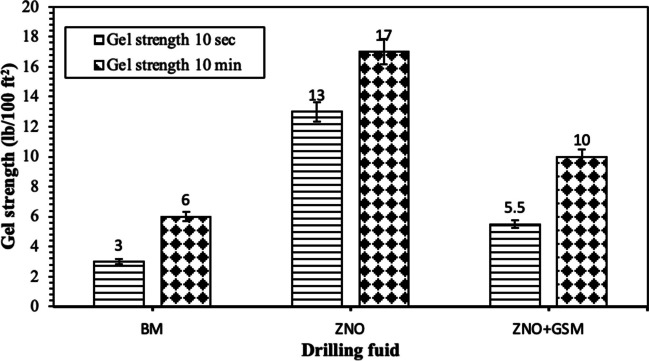
The findings of the BM, DNF, and NBDFs’ gel strengths at 10 s and 10 min

A DNF was created by introducing 1 wt% of the synthesized zinc ZNRs into the BM. This addition of synthesized zinc ZNRs significantly altered the rheological properties, as illustrated in Figs. 8 and 9. Notably, the apparent viscosity and yield point of the DNF remained constant at 13 cP and 7 lb/100 ft^2^, respectively, competed to the BM. However, by adding 1 wt% zinc ZNRs to the BM, the plastic viscosity enhanced from 7 to 13 cP (Fig. 10). Moreover, the gel strength experienced a substantial increase with the presence of the DNF (Fig. 11). The initial gel strength rose from 3 to 13 lb/100 ft^2^ when the ZNRs were mixed into the BM. Furthermore, the ZNR drilling fluid exhibited a notable 65% enhancement in the final gel strength recorded.

- Nano-biodegradable drilling fluid

Figures 10 and 11 present the rheological measurements of the NBDF (GS-ZNR). This fluid was formulated by combining the synthesized zinc ZNRs into the BDF containing GSM (75 µm and 1 wt%), comprising 1 wt% of 75 µm *gundelia* seed shell powder. A comparison with the BM and DNF reveals significant alterations across all determined rheological properties. The plastic viscosity of the GS-ZNR remained consistent at 7 cP in competed to the BM, but reduced by 46.15% when competed with the DNF. Conversely, the apparent viscosity slightly reduced from 13 to 11.5 cP. However, the GS-ZNR heightened the yield point from 7 to 9 lb/100 ft^2^, assessing both the BM and DNF (refer to Fig. 10). Furthermore, combining the optimum concentration of ZNRs with a 1 wt% concentration of GSM into the BM followed in an enhancement in both initial and final gel strengths, measuring 5.5 and 10 lb/100 ft^2^, respectively (Fig. 11). This raise is in comparison to the BM while showing a decrease compared to the DNF (Raza et al. 2023; Olaniyan and Sarah 2024).

### Rheological modelling

Table 4 shows the values of dial readings, shear rates, shear stress, and apparent viscosity parameters dependent based on different speeds of FANN rotation. The same approach is used in plotting the rheological behavior of the shear stress versus shear rate and the viscosity versus shear rate which provides a detailed understanding of how the shear rate and shear stress are derived for evaluating the rheological properties of drilling fluids.

**Table 4:** Rheological estimations of the developed drilling fluids

FANN speed (ϴ)	Dial reading	Shear rate (s^−1^)	Shear stress (dynes/cm)	Apparent viscosity (cP)
600	26	1021.92	184.6	18.06404
300	19	510.96	134.9	26.40128
200	16	340.64	113.6	33.34899
100	13.5	170.32	95.85	56.27642
6	9.5	10.^2192^	67.45	660.0321
3	9	5.1096	63.9	1250.587

**Fig. 12:**
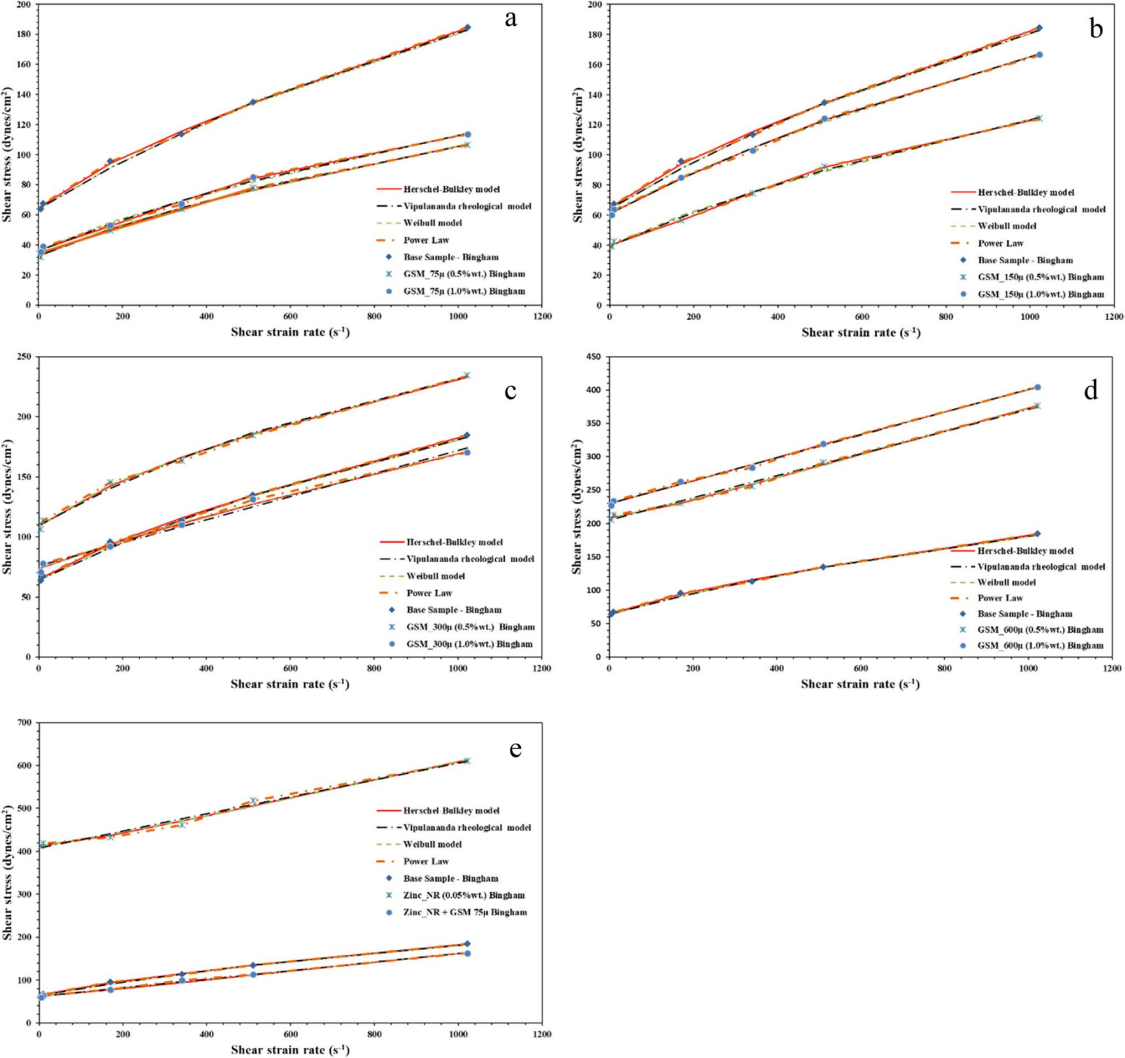
Shear strain versus shear stress relationship of the developed drilling fluids; **a** biodegradable at 75 µm particle size, **b** biodegradable at 150 µm particle size, **c** biodegradable at 300 µm particle size, **d** biodegradable at 600 µm particle size, and **e** DNF and NBDF

Figure 12 represents the relationship between shear rates and stresses across various drilling fluids, revealing different behaviors controlled by their specific compositions. Test results were compared against different rheological models such as Bingham, Power Low, Weibull, Herschel-Bulkley, and Vipulananda. Beginning with the BM, it shows that higher shear stress becomes apparent as the shear rate raises beginning 0 to 200 dynes/cm^2^. For drilling fluids created with different particle sizes of GSM, the smallest particle size of 75 µm displayed the lowest shear stress and shear rate correlation, dropping to 113 dynes/cm^2^ compared to the BM (Tahr et al. 2022a). At higher concentrations (1 wt%), there was a slight increase in shear stress. Similarly, for particle sizes of 150 µm, the shear rate and shear stress relationship remained in the range of 124 to 166 dynes/cm^2^. However, irregular shear stress values were noted for GSM with particle sizes of 300 and 600 µm, recording values of 234 and 404 dynes/cm^2^, respectively. Introducing nanorods notably increased shear stress, reaching a peak of 610 dynes/cm^2^. However, when combined with biodegradable materials, the shear stress decreased to 163 dynes/cm^2^, indicating a thinning performance of the drilling fluids. This thinning behavior is crucial for effective gel formation in stable situation and facilitates seamless flow with minimal pressure loss in cuttings transportation to surface. Remarkably, NBDFs, formed by blending GSM at 75 µm with ZNRs, exhibited exceptional thinning performance, highlighting their potential in drilling applications.

**Fig. 13:**
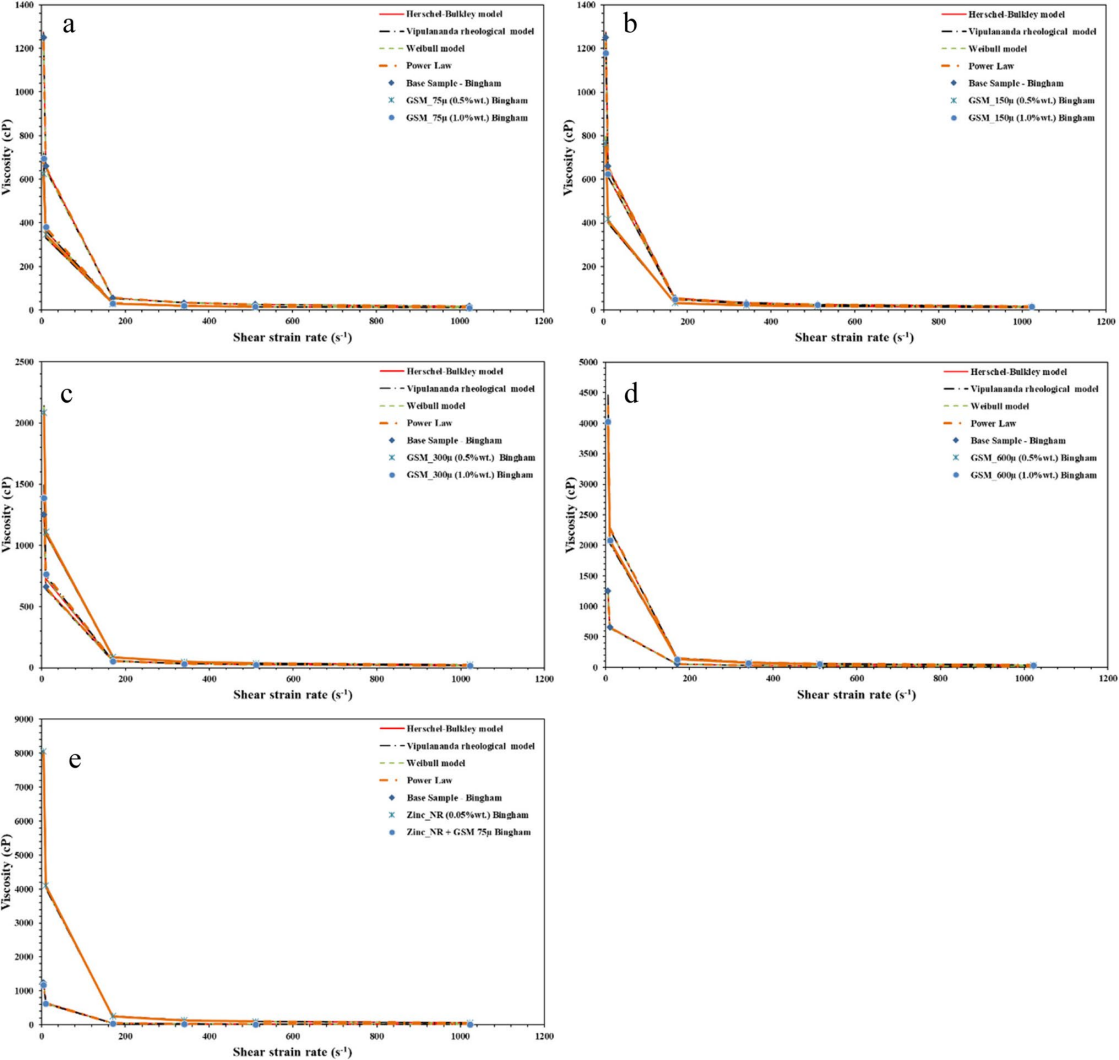
Shear strain versus viscosity relationship of the developed drilling fluids; **a** biodegradable at 75 µm particle size, **b** biodegradable at 150 µm particle size, **c** biodegradable at 300 µm particle size, **d** biodegradable at 600 µm particle size, and **e** DNF and NBDF

In addition, the relationship between shear rates and viscosity in various drilling fluids, each influenced by their unique compositions, is shown in Fig. 13. These behaviors were analyzed using different rheological models, including Bingham, Power Law, Weibull, Herschel-Bulkley, and Vipulananda, to highlight their differences. Shear-thinning characteristics are confirmed by the viscosity functions of shear rates, as seen in the shear stress plots. The GMS samples demonstrate a reduction in viscosity with increasing shear rates, while for BM, the viscosity remains nearly constant, indicative of its Newtonian nature. The extent of shear thinning in GMS samples varies with particle size and concentration; larger sizes and higher concentrations lead to a more noticeable drop in viscosity (Tahr et al. 2022b). At various temperatures, GMS samples exhibit higher viscosities than BM samples. This could be due to the nano-sized particles in the GMS samples, which enhance the fluid’s viscosity and stability. Additionally, the decrease in viscosity at higher shear rates is beneficial for drilling operations as it lowers pumping pressures and reduces frictional losses.

### Filtration properties

**Fig. 14:**
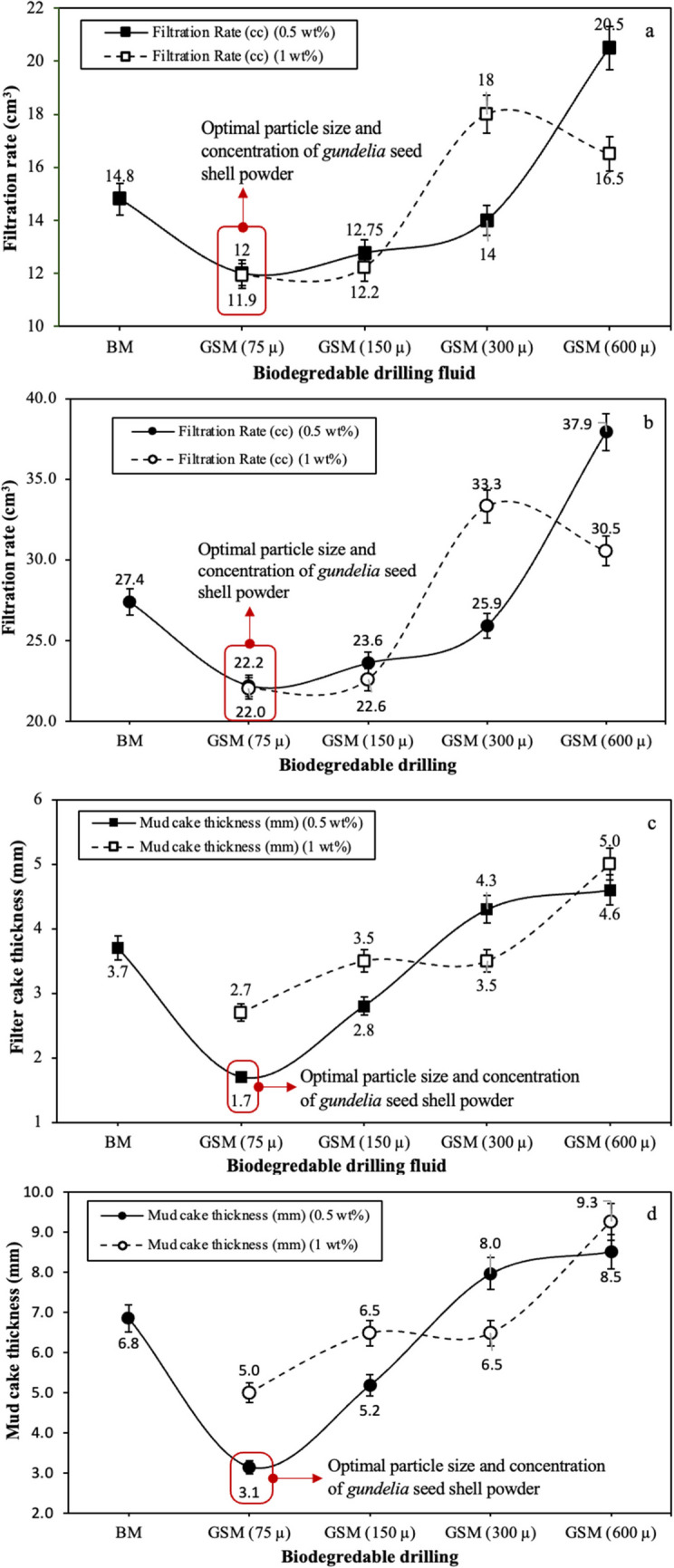
Filtration properties of the BDFs compared with the biodegradable once at different particle sizes and concentrations: **a** LTLP filtration rate, **b** HTHP filtration rate, **c** LTLP filter cake thickness, and **d** HTHP filter cake thickness

The filtration characteristics of the BM modified with ZNRs and waste materials were investigated at two condition such as LTLP and HTHP as shown in Fig. 14. The filtration rate of the BM is reduced when the *gundelia* seed shell powder with the particle size of 75 µm was added from 14.8 to 12 cm^3^. Afterwards, the rate of the filtration is increased with increasing the particle size of the used waste powder. The GSM with particle size 600 µm and concentration of 0.5 wt% of the *gundelia* seed shell has the highest filtration rate of 20.6 cm^3^. In general, the BDFs, derived from gundelia seed powder with various particle sizes, demonstrated optimal performance at 75 µm by effectively reducing the filtration rate through the formation of a substantial, impermeable filter cake. Meanwhile, the filtration rate of the GSMs prepared from mixing 1 wt% of the *gundelia* seed shell at different particle sizes within the BM is less compared with the 0.5 wt% expect 300 µm GSM (see Fig. 14a). The measurement of the filter cake thickness under LTLP conditions served as a vital parameter for filtration assessment across varying concentrations and particle sizes of both the BM and BDFs (see Fig. 14c). As can be seen, BM without the presence of the *gundelia* waste provided a filter cake of 3.7 mm in thickness. The *gundelia* seed shell in the concentration of 0.05 wt% created thinner filter cake when mixed with the BM for all prepared particle sizes expect 300 µm compared with 1 wt%. The thickest filter cake measured when 0.5 wt% *gundelia* waste with the particle size of 75 µm is added into the BM, which is 1.7 mm. Alternatively, the filter cake thickness was changed to 2.8, 4.3, and 4.6 mm when 0.5 wt% of the GSM at 150, 300, and 600 µm was added into the BM. However, the values are varied when 1 wt% of the GSM at 75, 150, 300, and 600 µm was added into the BM, which are 2.5, 3.5, 3.5, and 5, respectively.

The effect of pressure and temperature on the filtration properties of the BDFs prepared from *gundelia* waste at different particle sizes and concentrations can be observed on Fig. 14b and d. As is obvious, when the temperature and pressure are increased to 75 °C and 200 psi, respectively, the fluid loss is increased, and thicker mud cake is generated. For instance, the filtration rate of the BM is increased from 14.8 to 27.4 cm^3^ and the filter cake thickness is improved from 3.7 to 6.8 mm. The mentioned values of the filtration rate obtained by BM is decreased 22.2 and 22 cm^3^ when the *gundelia* waste with the particle size of 75 µm is included at concentrations of 0.5 and 1 wt%, correspondingly. Afterwards, the rate of fluid loss is increased with increasing the particle size of the biodegradable powder to the highest value of 37.9 cm^3^ by 0.5 wt% of GSM 600 µm. In terms of the concentration, the effect of the 1 wt% is remained slightly higher for all the particle sizes, except 300 µm, compared with the 0.5 wt% when the temperature and pressure adjusted to 75 °C and 200 psi, respectively (see Fig. 14b). In addition, the filter cake thicknesses of the BDFs (GSMs) at HTHP are presented on Fig. 14d. The thickness value of the filter cakes is almost doubled for all cases of the drilling fluids without and with the occurrence of the *gundelia* waste at altered particle sizes and concentrations. The filter cake of the BM is thickened from 3.7 to 6.8 mm at HTHP condition. However, this thickness is dropped by 54.4 and 23.5% under the effect of 0.5 wt% of the *gundelia* waste with the particle sizes of 75 and 150 µm, respectively, and increased by 17.6 and 36.7 with the particle sizes of 300 and 600 µm. Meanwhile, when the concentration of the waste powder rose to 1 wt%, the impact of the biodegradable on the reduction of the filter cake thickness is minimized and the lowest value of 5 mm is achieved with the particle size of 75 µm. Noticeably, the particle size of 300 µm in all cases of testing exhibited strangely and is out of the recorded trends of the measurements. Hence, the particle size of 75 µm is selected as the optimal value for the preparation of the BDFs from the *gundelia* seed shell waste. The enhanced performance of *Gundelia* seed shells with 75 µm in filtration can be attributed to their unique structural and chemical properties. *Gundelia* seed shells contain a high concentration of fibrous materials, including cellulose, hemicellulose, and lignin. These fibrous components provide a high surface area and porosity, which enhance their ability to adsorb and retain particles, effectively reducing the permeability of the filter cake and minimizing fluid loss (Li et al. 2024a, b). The particle size of 75 µm is particularly effective for bridging the pores and microfractures within the formation. This bridging action helps to create a more compact and impermeable filter cake on the wellbore wall, which prevents further invasion of the drilling fluid into the formation (Meza et al. 2017). Additionally, the natural composition of *Gundelia* seed shells includes various organic compounds, such as oils and waxes, which enhance the lubricity of the drilling fluid. This increased lubricity reduces the friction between particles and facilitates the formation of a thin, uniform, and resilient filter cake. The presence of these compounds also helps in stabilizing the fluid loss control properties of the drilling fluid under different temperature and pressure conditions (Zhao et al. 2022).

**Fig. 15:**
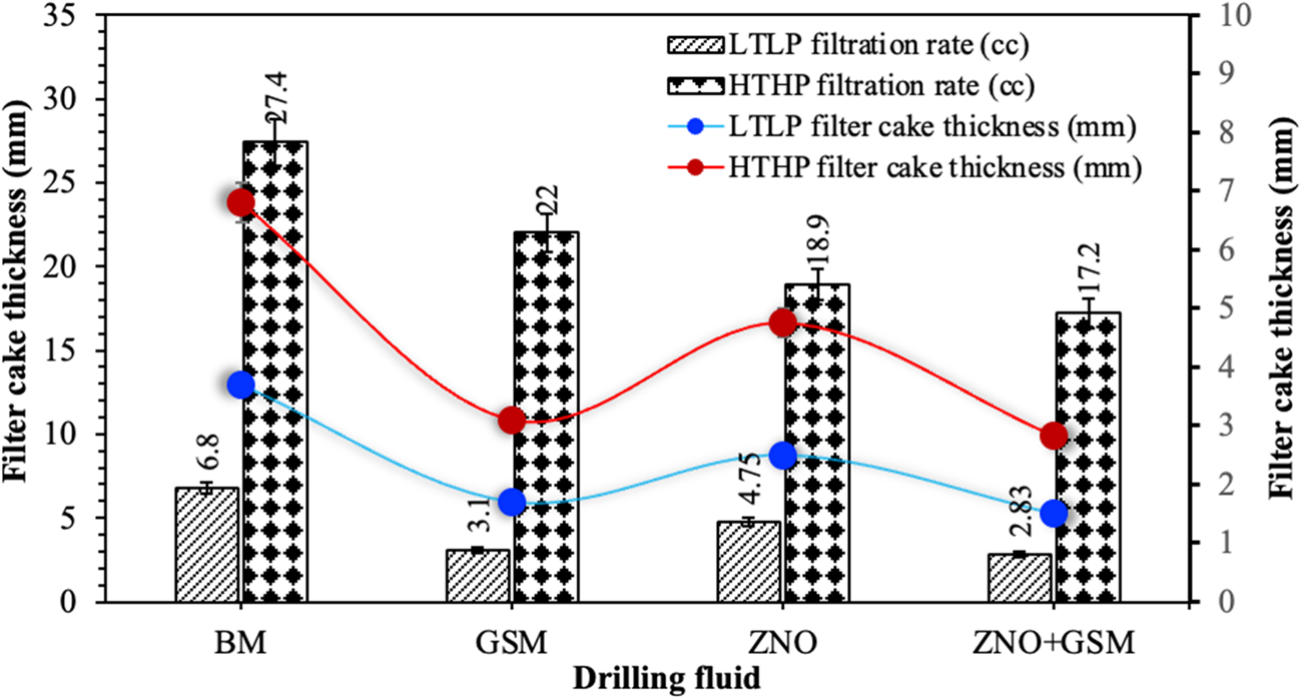
Filtration properties of the BM, optimal BDF, DNF and NBDF under LTLP and HTHP testing conditions

Figure 15 shows the filtration properties of the drilling fluids. The filter cake thickness of the drilling fluid started to reduce by 54 and 32.4% when the recipe of the mud is changed from the bentonite water-based mud to the BDF and DNF, respectively, under the LTLP condition. Meanwhile, further reduction of additional 5.45% was noticed when the NBDF is used at the same testing condition but with mixing 1 wt. ZNRs and GSM at 75-µm particle size. The values of the mud cake thicknesses for all mentioned types of the drilling fluids are higher at HTHP condition compared with the LTLP. At the same time, the filtration rate exhibited the same behavior under the impact of temperature and pressure; when these two factors are increased, the rate of fluid loss is increased as well. As can be seen, the filtration rate is significantly reduced when the recipe of the drilling fluid is changed from the BM to GSM, ZNR, and ZNO + GSM drilling fluids (see Fig. 14). The influence of the DNF is lowest compared with the optimal biodegradable and NBDFs but it enables additional decrease of the filtration rate when mixing with the optimal biodegradable one (Oni et al. 2023; Ghazali et al. 2024). The minimum rate of the filtration is obtained by a NBDF created from combining 1 wt% zinc ZNRs and *gundelia* seed shell powder at 75-µm particle size within the BM at ambient testing condition, which is 2.83 cm^3^. However, 17.2 cm^3^ is the lowest filtration rate recorded under HTJP testing condition for the same drilling fluid.

The specific role of the nanorod structure of ZNRs in the filter cake buildup phenomena can be attributed to several factors related to their unique shape and properties. The high surface area to volume ratio of the nanorods allows for more effective interaction with the particles in the drilling fluid and the formation, enhancing adsorption and trapping of particles, thus contributing to a denser and more impermeable filter cake (Oseh et al. 2023). The elongated shape of ZNRs enables effective bridging across pores and fractures, blocking flow paths and forming a compact, robust filter cake that minimizes fluid loss (Vryzas and Kelessidis 2017). Additionally, the rigid structure of ZNRs provides mechanical reinforcement to the filter cake, increasing its resistance to pressure and mechanical degradation (Tang et al. 2020). The surface chemistry of ZNRs, influenced by the phytochemicals used in their synthesis, improves compatibility with drilling fluid components, leading to better dispersion and integration within the filter cake (Bardhan et al. 2024). Furthermore, the thermal stability of ZNRs, maintained by stabilizing agents from the plant extracts used in their synthesis, helps preserve the integrity of the filter cake under high-temperature conditions encountered during drilling (Ikram et al. 2021). These factors collectively contribute to the formation of a more effective and resilient filter cake, essential for minimizing fluid loss and maintaining wellbore stability.

**Fig. 16:**
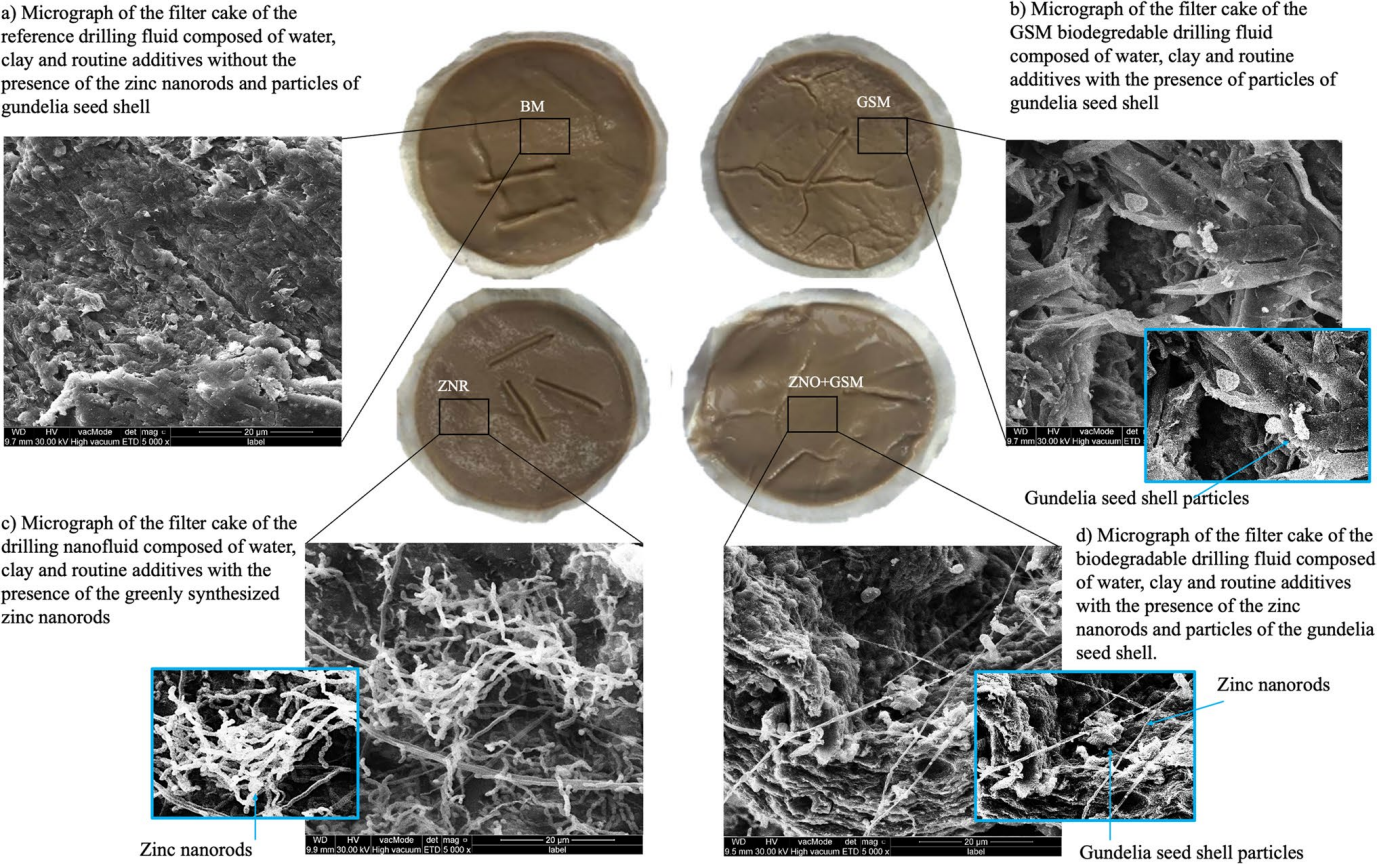
Snapshoots and micrographs of the filter cakes resulted from **a** BM, **b** BDF, **c** DNF, and **d** NBDF

Figure 16 demonstrates the snapshoots and micrographs of the filter cakes obtained from the filtration measurements. As is clear, a small variation in the color of the filter cakes can be noticed due to the change in the composition of the studied drilling fluids. Meanwhile, a high difference is observed between the SEM micrographs of the filter cakes. The graph shown in Fig. 14a illustrates the structure of the filter cake surface produced from the BM without the presence of the ZNRs and *gundelia* waste particles, while the particles of the *gundelia* waste are obvious of the filter cake surface created by the BDF (see Fig. 16b). In addition, the darker filter cake was produced by the DNF with the presence of the nanorods on its surface as shown in Fig. 14c. Furthermore, when both the synthesized ZNRs and the *gundelia* seed waste particles were mixed within the BM, their presence on the resulted filter cake is clear (see Fig. 16d). Overall, the NBDF provided the thinnest filter cake with less fluid loss.

## Performance evaluation of the current work compared with the literature

A comparative evaluation of the result outcomes of this study with the recently published works related to the application of bio-based substances in improving the main properties of drilling fluid including rheology, fluid loss, and filter cake thickness is shown in Table 5. Different studies were included from various parts of the world using different materials. As can be seen, Novara et al. (2021) used two types of nanoparticles (SiO_2_ and Al_2_O_3_) to improve the rheology and filtration of the freshwater-based drilling fluid. Their results shown that silica was more effective in reducing the rate of fluid loss by 26.9% and thickness of the filter cake by 23.8% compared with the alumina which is left negative impact. Meanwhile, alumina provided a better performance in improving the rheological properties of the drilling fluid. In 2023, a group of researchers from Malaysia developed a bio-based graphene from the oil palm and added to the water-based drilling fluid at 1 wt% concentration (Safian et al. 2023). They conducted their experiments of filtration and rheology measurements at 120 °C and 200 psi and reported that the filtration rate reduced by 41.9% and decreased the thickness of the filter cake by 53.8% along with leaving a weak impact on the rheological behavior. In addition, eggshell (reported as eggshell nanoparticles (NPs)) as the waste substance at the ultrafine size (5 µm) was used at the concentration of 6 lb/bbl (Fadairo and Oni 2024). They reported that the eggshell NPs enabled 17% and 15.6% reduction in the filtration rate and thickness of filter cake, respectively, under 200 °C and 400 psi condition. Furthermore, the mechanism used in this study was the most effective one compared with the mentioned studies in terms of decreasing the fluid loss and the filter cake thickness by 58.38% and 55%, respectively. Although the impact of both used environmentally friendly substances were moderate on rheological properties but the main purpose was modification of the filtration properties. Overall, the idea of the development of nano-biodegradable drilling fluid from green ZNRs and *gundelia* waste is new and effective in terms of reducing the toxicity of the drilling mud circulation and drilling mud waste which meets current environmental standards and reduces the cost of the mud pit remediation (Martin et al. 2023; Liu et al. 2023). However, for future research, the following pathways are recommended: Conduct systematic studies to determine the optimal concentration and ensure uniform dispersion of ZNR in the drilling fluid. Investigate various surface modifications and coatings for ZNR to enhance their compatibility and interaction with the drilling fluid components. Synthesize ZNR with different sizes and aspect ratios to identify the most effective configurations for enhancing drilling fluid properties. Additionally, study the interactions between ZNR and other common drilling fluid additives to identify potential synergistic or antagonistic effects and optimal formulations.

**Table 5:**
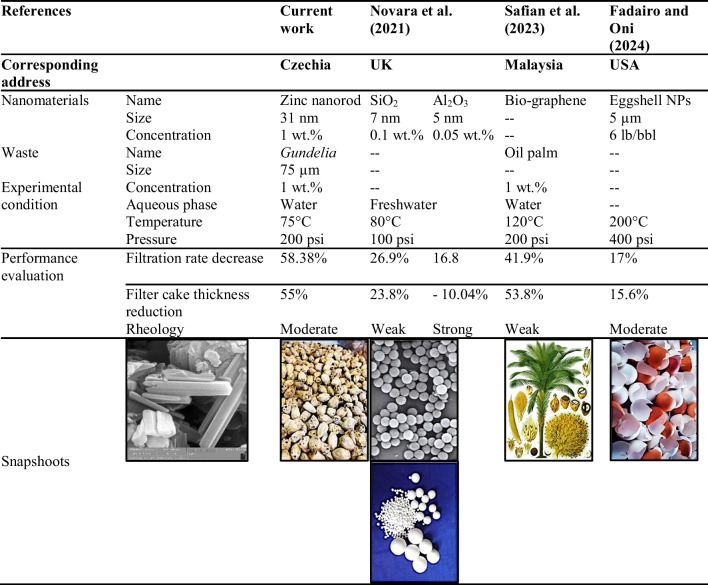
Summary of the performance evaluation of the natural surfactants prepared in this study compared with the previously reported once

## Conclusions

The aim of this study revolves around creating a sustainable NBDF that minimizes fluid loss, produces the thinnest mud cake, and enhances rheological properties. This innovative drilling fluid is crafted using locally sourced waste from gundelia seeds and synthesized ZNRs extracted from the *Cydonia oblonga* plant. Evaluation was based on the performing of the BM, adhering to API-SPEC-13A-2010 standards. The study’s findings can be summarized as follows:

- The analytical methods of Uv–Vis, SEM, DLS, and FTIR confirmed the validity of the synthesized nanorods.
- The smaller particle size of the *gundelia* seed waste is more effective in enhancing the drilling fluid performance compared with the larger ones, and the particle size of 75 µm is detected as the optimal once.
- Among both 0.5 and 1 wt% concentrations of the *gundelia* seeds waste, only slight change is observed in drilling fluid properties.
- The drilling fluid properties were affected by the DNF obtained from the ZNRs, although the impact was not as noteworthy as that from the waste of *gundelia* seeds.
- The developed NBDF enabled modification in the values of the plastic viscosity, apparent viscosity, gel strength, and yield point to a favorite range. Thus, better share rate and shear strain relation was achieved.
- The created NBDF reduced the thickness of the filter cake and fluid loss volume by more than 50% competed with the BM.
- HTHP has a direct relationship with the drilling fluid filtration properties and increased filter cake thickens and filtration rate.

## Data Availability

The datasets used and analyzed during the current study are available from the corresponding author on reasonable request.
